# Chemodiversity in Selaginella: a reference system for parallel and convergent metabolic evolution in terrestrial plants

**DOI:** 10.3389/fpls.2013.00119

**Published:** 2013-05-10

**Authors:** Jing-Ke Weng, Joseph P. Noel

**Affiliations:** Howard Hughes Medical Institute, Jack H. Skirball Center for Chemical Biology and Proteomics, The Salk Institute for Biological StudiesLa Jolla, CA, USA

**Keywords:** *Selaginella*, specialized metabolism, chemodiversity, parallel evolution, convergent evolution

## Abstract

Early plants began colonizing the terrestrial earth approximately 450 million years ago. Their success on land has been partially attributed to the evolution of specialized metabolic systems from core metabolic pathways, the former yielding structurally and functionally diverse chemicals to cope with a myriad of biotic and abiotic ecological pressures. Over the past two decades, functional genomics, primarily focused on flowering plants, has begun cataloging the biosynthetic players underpinning assorted classes of plant specialized metabolites. However, the molecular mechanisms enriching specialized metabolic pathways during land plant evolution remain largely unexplored. *Selaginella* is an extant lycopodiophyte genus representative of an ancient lineage of tracheophytes. Notably, the lycopodiophytes diverged from euphyllophytes over 400 million years ago. The recent completion of the whole-genome sequence of an extant lycopodiophyte, *S. moellendorffii*, provides new genomic and biochemical resources for studying metabolic evolution in vascular plants. 400 million years of independent evolution of lycopodiophytes and euphyllophytes resulted in numerous metabolic traits confined to each lineage. Surprisingly, a cadre of specialized metabolites, generally accepted to be restricted to seed plants, have been identified in *Selaginella*. Initial work suggested that *Selaginella* lacks obvious catalytic homologs known to be involved in the biosynthesis of well-studied specialized metabolites in seed plants. Therefore, these initial functional analyses suggest that the same chemical phenotypes arose independently more commonly than anticipated from our conventional understanding of the evolution of metabolism. Notably, the emergence of analogous and homologous catalytic machineries through convergent and parallel evolution, respectively, seems to have occurred repeatedly in different plant lineages.

## INTRODUCTION

**FIGURE 1:**
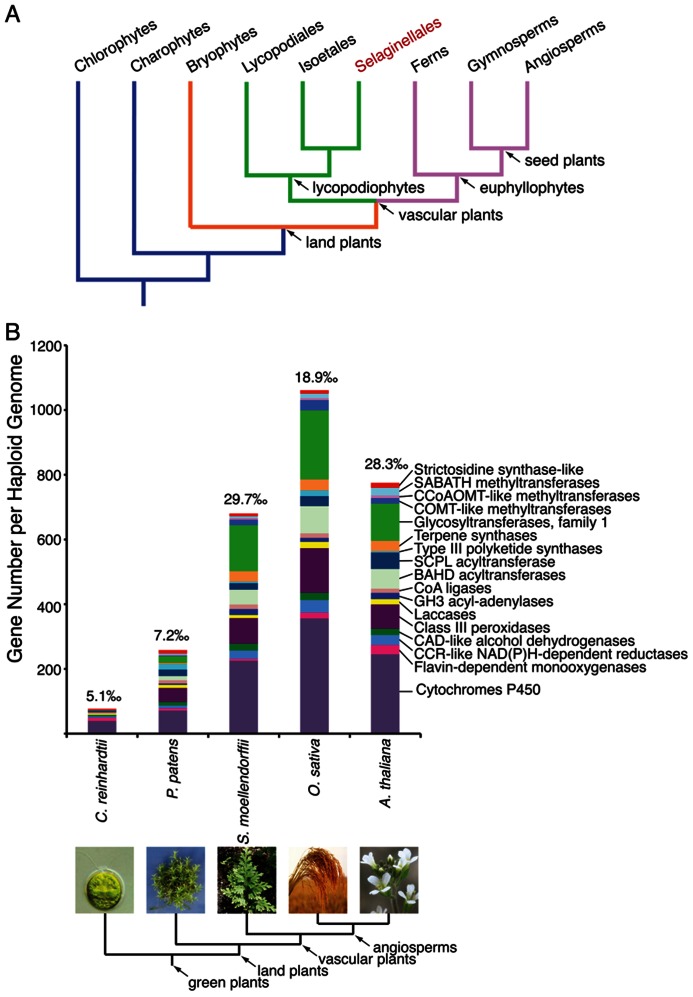
**(A)** A simplified cladogram illustrating the phylogeny of the green plant lineage. *Selaginella* is the only genus under the order Selaginellales (highlighted in red), and represents an ancient lineage of vascular plants, lycopodiophytes. **(B)** A representative expansion of enzyme families implicated in specialized metabolism during plant evolution. The gene number for each enzyme family counted per haploid genome from five species representative of the green plant lineage is shown. The proportion of the sum of these genes in relation to the haploid gene complement is denoted on top of each bar. Due to our currently limited knowledge of the temporal expression and biochemical function of specialized metabolic enzymes, the enzyme families and numbers of members listed likely underestimate the true biosynthetic potential of any green plant.

*Selaginella*, also known as spikemoss, is the only surviving genus within the Selaginellaceae family. *Selaginella* includes more than 700 species widely distributed around the globe (Little et al., 2007). Selaginellaceae, together with the other two extant families Lycopodiaceae (clubmosses) and Isoetaceae (quillworts) within the division Lycopodiophyta, comprise the oldest lineage of vascular plants surviving on earth (Banks, 2009). Fossil records suggest that lycopodiophytes, often referred to as lycophytes, diverged from all other vascular plants including ferns and seed plants (euphyllophytes) more then 400 million years ago (Pryer et al., 2004; **Figure 1A**). Lycophytes dominated the earth flora during the Carboniferous period encompassing a tremendous expansion of terrestrial life roughly 360 million years ago (Stewart and Rothwell, 1993; Banks, 2009).

Unlike extant lycophytes, which are typically small in stature, many extinct lycophytes, such as the celebrated lepidodendrales (scaled trees), grew to enormous heights (Stewart and Rothwell, 1993). Those giant lycophytes formed vast swamp forests, resulting in an interval of tremendous carbon fixation by terrestrial life, precipitating a significant drop of atmospheric CO_2_ levels during the late Paleozoic era (Berner, 1993; Berner et al., 2000). Indeed, 70% of the biomass responsible for the Bashkirian and Moscovian coal formations in Euramerica came from lycophytes (Manfroi et al., 2012). As one of the few lycophyte genera that survived the Permian–Triassic extinction event, *Selaginella* has been a longstanding subject of investigation for botanists and paleontologists. The recent completion of the whole-genome sequence of *S. moellendorffii* now opens new avenues for integrating (paleo)botany with comparative plant genomics, development, and biochemistry to better understand the evolution and adaptation of terrestrial plants to a myriad of challenging ecosystems (Banks, 2009; Banks et al., 2011).

Plants began their migration from an aquatic existence onto land during the mid-Ordovician period approximately 450 million years ago, and over the ensuing 40 million years spread across the terrestrial earth. In addition to key developmental innovations, early terrestrial plants began the extensive elaboration of specialized metabolic networks (**Figure 1B**). These systems, rooted in core metabolism, biosynthesize a plethora of chemicals, often known as “secondary” metabolites, as adaptive strategies to challenging biotic and abiotic ecological pressures (Weng et al., 2012). Many of these chemicals, e.g., cuticular components and phenolic compounds, are ubiquitous in all extant land plants, providing essential chemical protectants against desiccation and UV radiation. Other specialized metabolites, including those that constitute colors, flavors, and scents, often occur in a lineage-specific manner, playing specialized roles for the host species in their unique ecological niches (Weng et al., 2012).

Our current understanding of plant specialized metabolism and its evolutionary underpinnings has been primarily driven by studying flowering plants, ranging from well-established model species, e.g., *Arabidopsis* and rice (Romeo, 2004; D’Auria and Gershenzon, 2005), to reference species including medicinal plants with notable pharmacological properties, e.g., Madagascar periwinkle and opium poppy (Facchini and De Luca, 2008; De Luca et al., 2012). These studies revealed tremendous chemodiversity in flowering plants, echoing their extensive speciation and global domination over the last 170 million years following the Permian–Triassic extinction event (Wikstrom et al., 2001).

Probing chemodiversity and its underlying specialized metabolic systems in *Selaginella*, a genus that diverged from all euphyllophytes over 400 million years ago, should accelerate our systematic and integrated understanding of plant metabolic evolution over a much longer time scale. The inclusion of a phylogenetic diversity of reference plant species representing a more complete genomic and metabolic record of terrestrial life in plant biology promises to illuminate how metabolic evolution shaped the remarkable adaptability and biodiversity seen in terrestrial plants living today (**Figure 1B**). This rapidly expanding molecular understanding of plant adaptation over the last 450 million years of varied climates also portends a future where our ability to predict and fine-tune plant fitness in the face of global climate change will serve as an essential component in the sustainability of the global food chain.

In addition, as a genus with a global distribution pattern, *Selaginella* has long been recognized for its pharmacological activities as evidenced by its extensive use by indigenous cultures in herbal medicines and tonics. The earliest documentation of *Selaginella*-based treatments appeared in Shen Nong Ben Cao Jing (The Divine Farmer’s Materia Medica) in 2737 BC, where *Selaginella* was used to treat inflammation, amenorrhea, and abdominal lumps in women (Yang and Flaws, 1998). *S. bryopteris* or sanjeevani (one that infuses life) has been used for centuries in Indian ayurvedic medicine to treat burning urination, menstrual irregularities, and jaundice (Sah et al., 2005). Despite a long history of *Selaginella* being used as an herbal remedy and tonic, the scientific basis underpinning its efficacy in treating various maladies is lacking. Over the last several decades, the isolation and structural elucidation of natural chemicals from the *Selaginella* genus has expanded and several compounds are now being tested for pharmacological efficacy using established protocols (Setyawan, 2011). The documented record of the *Selaginella* genus as a source of medicinal plants, when complemented by genomics, metabolomics, and drug discovery, will serve as a foundation for unanticipated breakthroughs in the development of therapeutic and disease prevention agents from a currently understudied medicinal plant family.

This overview of a currently underappreciated plant species should provide readers with an accessible and up-to-date reference of specialized metabolites and their associated biosynthetic pathways identified in the *Selaginella* genus. We also hope that some of the concepts regarding *Selaginella* small molecule biosynthesis, drawn from comparative genomics and initial gene annotation, will stimulate more in-depth functional studies of the evolutionary and biochemical mechanisms of metabolism in the green plant lineage. More specifically, an argument can be made for technology development to ultimately move *Selaginella* from its current role as a reference species to a future model system.

Here 130 natural products, previously reported from *Selaginella*, were sorted into six of the major categories of plant specialized metabolites including flavonoids, lignans, selaginellins, other phenolics, alkaloids, and terpenoids. Moreover, by integrating chemotaxonomic, phylogenetic, and enzymological information drawn from multiple plant genomes into a contemporary understanding of plant metabolism, we posit plausible biosynthetic routes through which different classes of specialized metabolites might be biosynthesized in *Selaginella*.

Throughout this review, convergent evolution is separated from parallel evolution. This is possible as protein folds associated with specific biochemical activities can often be unequivocally established. By using homologous protein structures as precise characteristics delineating descent from a common ancestral fold, parallel and convergent evolution are explicitly separated (Zhang and kumar, 1997). When ancestral descendants possessing distinct biochemical activities but a shared structural lineage nevertheless contemporarily evolve to synthesize the same metabolite, the term parallel evolution is used. When distinct protein structures sharing no structural similarity result in the synthesis of the same metabolite, the term convergent evolution is employed.

**FIGURE 2:**
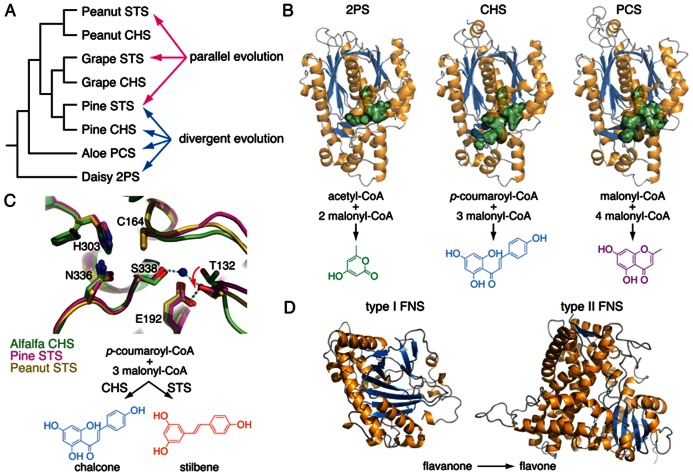
**Examples of divergent, parallel, and convergent evolution in plant specialized metabolism**. **(A)** A cladogram showing the phylogenetic relationship of several land plant type III polyketide synthases (PKSs) involved in specialized metabolism. Whereas the expansion of enzyme families by duplication and selection over time leads to functional divergence among descendants, identical biochemical functions do arise independently from homologous ancestral forms through parallel evolution. 2PS, 2-pyrone synthase; CHS, chalcone synthase; PCS, pentaketide chromone synthase; STS, stilbene synthase. **(B)** Divergence of three plant type III PKSs, 2PS, CHS, and PCS. Mutational trajectories reshape the volume of the active sites (green surface representation) directly correlated with divergent biochemical functions. **(C)** An overlay of the active sites of CHS from alfalfa (green) and two STSs from pine (magenta) and peanut (yellow), respectively. Parallel evolution of STSs from CHSs in several plant lineages involves distinct mutational trajectories in otherwise homologous three-dimensional structures. These mutations at disparate positions in the STSs’ primary sequences lead to the same conformational shifts of Thr132 in the active sites (red arrow). These separate alterations of the same structural component dictate a switch from cyclization by Claisen condensation in CHSs to cyclization by aldol condensations in STSs. **(D)** Convergent evolution of flavone synthases (FNS) in plants. Whereas most flowering plants examined to date contain type II FNSs belonging to the cytochrome P450 enzyme family, plants in the Apiaceae employ type I FNSs belonging to the non-homologous 2-oxoglutarate-dependent dioxygenase family.

During the course of genome analysis and the cataloging of the diversity of small molecules produced by *Selaginella* species, it became clear that many of the specialized metabolites have occurred repeatedly during land plant evolution. This observation extends our current understanding of the independent radiation of specialized metabolic enzyme families through parallel or convergent evolution of the biosynthesis of identical metabolites (**Figure 2**).

## FLAVONOIDS

**FIGURE 3:**
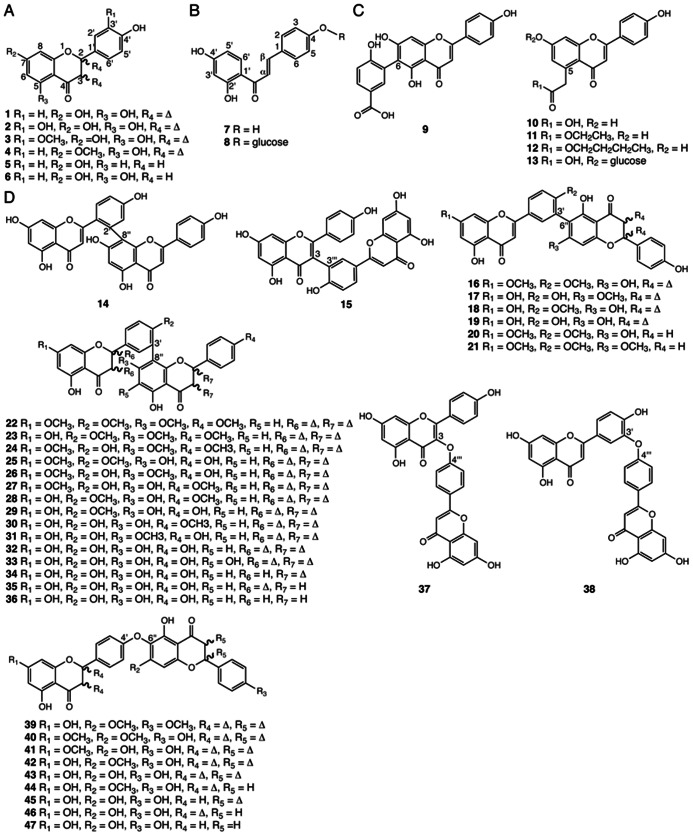
**Flavonoids identified from *Selaginella***. **(A)** Common flavones and flavanones: apigenin (**1**), luteolin (**2**), chrysoeriol (**3**), genkwanin (**4**), 4′,7-dihydroxyflavanone (**5**), 4′,5,7-trihydroxyflavanone (**6**). **(B)** Chalcones: 6′-deoxychalcone (**7**), 6′-deoxychalcone, 4-*O*-β-glucoside (**8**). **(C)** Flavones with unusual ring substitutions: 6-(2′-hydroxy-5′-carboxyphenyl)-apigenin (**9**), 5-carboxymethyl-4′,7-dihydroxyflavone (**10**), 5-carboxymethyl-4′,7-dihydroxyflavone ethyl ester (**11**), 5-carboxymethyl-4′,7-dihydroxyflavone butyl ester (**12**), 5-carboxymethyl-4′,7-dihydroxyflavone-7-*O*-β-D-glucopyranoside (**13**). **(D)** Biflavonoids: 2′,8^′′^-biapigenin (**14**), taiwaniaflavone (**15**), 7,4′-di-*O*-methylrobustaflavone (**16**), 7′-*O*-methylrobustaflavone (**17**), 4′-*O*-methylrobustaflavone (**18**), robustaflavone (**19**), 7,4′-di-*O*-methyl-2′,3′-dihydrorobustaflavone (**20**), 7,4′,7^′′^-tri-*O*-methyl-2′,3′-dihydrorobustaflavone (**21**), 7,4′,7^′′^,4^′′′^-tetra-*O*-methylamentoflavone (**22**), kayaflavone (**23**), heveaflavone (**24**), ginkgetin (**25**), 7,7′-di-*O*-methylamentoflavone (**26**), 4′,7′-di-*O*-methylamentoflavone (**27**), isoginkgetin (**28**), bilobetin (**29**), podocarpusflavone A (**30**), sostetsuflavone (**31**), amentoflavone (**32**), sumaflavone (**33**), 2,3-dihydroamentoflavone (**34**), 2′, 3′-dihydroamentoflavone (**35**), tetrahydroamentoflavone (**36**), delicaflavone (**37**), ochnaflavone (**38**), cryptomerin B (**39**), pulvinatabiflavone (**40**), 7-*O*-methyl-hinokiflavone (**41**), isocryptomerin (**42**), hinokiflavone (**43**), 2′,3^′′^-dihydroisocryptomerin (**44**), 2,3-dihydrohinokiflavone (**45**), 2′,3′-dihydrohinokiflavone (**46**), tetrahydrohinokiflavone (**47**).

Phenolic flavonoids are a widespread class of polyketide- and phenylpropanoid-derived specialized metabolites found in all land plants (Grotewold, 2006). Important flavonoids include anthocyanins, condensed tannins, and phlobaphenes. Many serve as UV sunscreens as well as important color cues for pollinators and seed dispersers particularly in flowering plants. Other flavonoids function as phytoalexins and antifeedants in plant defense against pathogens and herbivores, respectively (Winkel-Shirley, 2001). Certain flavonoids are also known to mediate signaling processes between plants and their symbiotic microbes (Hassan and Mathesius, 2012). Common flavones and flavanones with various hydroxy or methoxy substitutions at carbon positions 5, 7, and 3′ (**1–6**) are found in *Selaginella* species (Zheng et al., 2004b; Cao et al., 2009; Yang et al., 2010; Yobi et al., 2012; **Figure 3A**). The identification of 3′-hydroxylated or methoxylated flavones such as luteolin (**2**) and chrysoeriol (**3**) in *Selaginella* suggests that *Selaginella* must contain a flavonoid 3′-hydroxylase (F3′H) activity and a flavonoid 3′-*O*-methyltransferase (F3′OMT) activity. In flowering plants, F3′H and F3′OMT catalyze sequential reactions in anthocyanin biosynthesis, forming an important branch pathway in tuning flower color (Brugliera et al., 1999; Kim et al., 2006).

Interestingly, infrequently found 6′-deoxychalcone and its glucoside (**7**, **8**), as well as its cyclized flavanone derivative 4′,7-dihydroxyflavanone (**5**), which are often thought to be restricted to legumes, are also found in *S. doederleinii* (Yang et al., 2010; **Figures 3A, B**). In legumes, the production of 6′-deoxychalcone requires an additional chalcone reductase (CHR; Bomati et al., 2005), and the cyclization of the resultant product, 6′-deoxychalcone (**7**) to 4′,7-dihydroxyflavanone (**5**), requires specialized chalcone isomerases (CHIs). To date, these activities have only been found in the Fabaceae family (Maxwell et al., 1989). The presence of 4′,7-dihydroxyflavanone (**5**) in *Selaginella* suggests that *Selaginella* may contain a 6′-deoxychalcone-derived flavonoid biosynthetic pathway homologous or analogous to that in legumes.

Several flavonoids with unusual modifications were reported in *Selaginella* (**Figure 3C**). For example, 6-(2′-hydroxy-5′-carboxyphenyl)-apigenin (**9**) was isolated from *S. tamariscina* (Liu et al., 2010), and 5-carboxymethyl-4′,7-dihydroxyflavone (**10**) and its ethyl/butyl esters (**11**, **12**) were reported in *S. moellendorffii* (Zhu et al., 2008; Cao et al., 2009; Wang et al., 2011). Biosynthetically, compound **9** can be generated from radical coupling of apigenin and *p*-hydroxy benzoic acid. The core chemical scaffold of compounds **10**–**13** were recently proposed to be synthesized by an non-canonical chalcone synthase (CHS)-like type III polyketide synthase (PKS) capable of an additional 2-carbon extension resulting from the decarboxylative condensation of malonyl-CoA (Austin and Noel, 2003; Cao et al., 2009).

Besides monomeric flavonoids, *Selaginella* is a rich source for biflavonoids (Setyawan, 2011). Medicinally, biflavonoids associate with assorted pharmacological properties including antimicrobial, antiviral, anticancer, anti-inflammatory, and anti-fibrillogenesis activities (Ma et al., 2001; Tang et al., 2003; Pan et al., 2005; Setyawan, 2011; Thapa et al., 2011). Seven dimeric linkage types are found in biflavonoids isolated from *Selaginella*, including 2′–8″ (**14**), 3–3‴ (**15**), 3′–6″ (**16**–**21**), 3′–8″ (**22**–**36**), 3-*O*-4‴ (**37**), 3′-*O*-4‴ (**38**), and 4′-*O*-6″ (**39**–**47**; Lin et al., 1994, 2000; Silva et al., 1995; Lee et al., 1996, 2008, 2009; Sun et al., 1997; Ma et al., 2001, 2003; Kang et al., 2004; Cheng et al., 2008; Feng et al., 2008; Zheng et al., 2008, 2011; Zhu et al., 2008; Cao et al., 2009, 2012; Liu et al., 2010; Setyawan, 2011; Wu and Wang, 2011; Yang et al., 2011; Zhang et al., 2011, 2012a; **Figure 3D**). Although little is known about the mechanisms governing biflavonoid crosslinks in plants, biflavonoids likely dimerize via radical coupling reactions mediated by peroxidases (Yamaguchi and Kato, 2012), a catalytic reaction shared with lignan and lignin biosynthesis (Umezawa, 2003; Ralph et al., 2004).

Indeed, *S. moellendorffii* contains 79 annotated class III peroxidase-like sequences, accounting for 3.5‰ of the current gene number of the genome (Weng and Chapple, 2010; **Figure 1B**). These gene quantities rival those of several sequenced flowering plant species, and greatly exceed those of the basal bryophyte moss *Physcomitrella patens* (Weng and Chapple, 2010; **Figure 1B**). Presumably, a fraction of these class III peroxidase sequences encode functional enzymes catalyzing the regioselective coupling reactions central to the biosynthesis of structurally diverse biflavonoids in *Selaginella*.

*Selaginella* biflavonoids, particularly those containing the 3′–8″, 3′–6″, and 4′-*O*-6″ linkages, also are decorated by extensive structural elaborations including *O*-methylation, 2,3-desaturation of the naringenin unit, and 6″-hydroxylation in the case of the 3′–8″ linked sumaflavone (**33**; **Figure 3D**). The presence of these compounds indicates the involvement of catalytically divergent and convergent OMTs, hydroxylases, and flavone synthases (FNSs). It is noteworthy that flowering plants typically contain two structurally and catalytically convergent types of FNS (**Figure 2D**). In most of the plant species examined to date, the production of flavones from (2*S*)-flavanones is catalyzed by the membrane-bound cytochrome P450 FNS II. However, in Apiaceae, this reaction is catalyzed by a soluble type I FNS belonging to the 2-oxoglutarate-dependent dioxygenase family (Leonard et al., 2005). Homology-based searches using either of the convergent-derived angiosperm type I FNS or type II FNS sequences against the *Selaginella* genome failed to retrieve clear homologs of either type of FNS. It is possible that *Selaginella* contains a distinct type I or II FNS. A functional FNS may have evolved independently from those found in other vascular plants over a time period sufficient so that the extant sequences do not clearly clade with their counterparts in flowering plants. Alternatively, *Selaginella* FNSs may encompass a distinct lineage of either 2-oxoglutarate-dependent dioxygenases or cytochrome P450s through parallel evolution or yet another oxidase family through convergent evolution.

## LIGNANS

**FIGURE 4:**
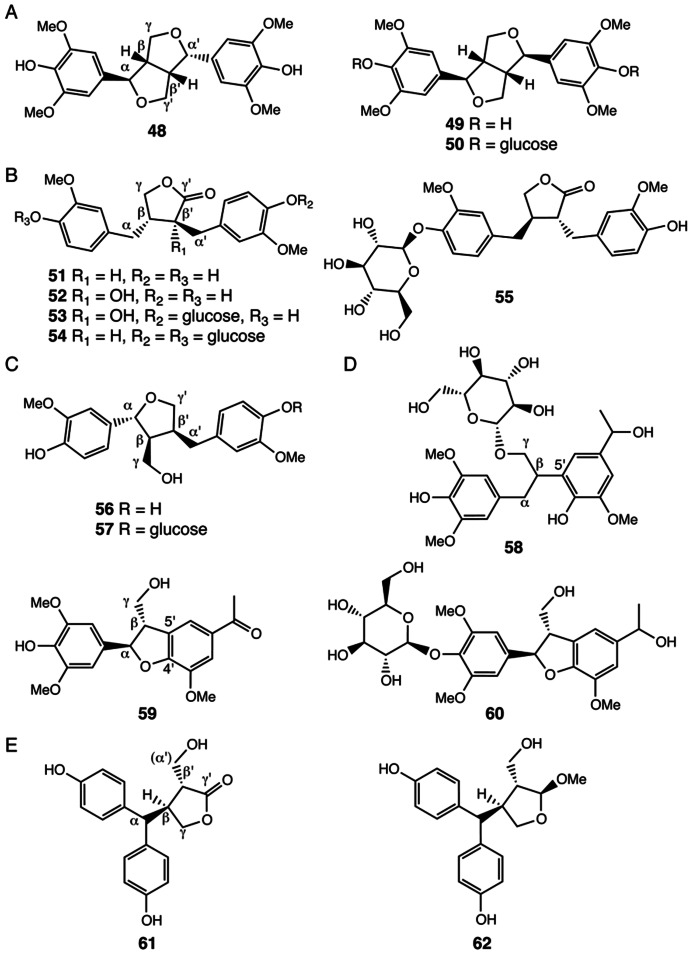
**Lignans identified from *Selaginella*.** **(A)** Lignans with the β-β′/ γ-*O*-α′/ α-*O*-γ′ linkage: (-)-lirioresinol A (**48**), (+)-syringaresinol (**49**), (+)-syringaresinol-4,4′-di-*O*-β-D-glucopyranoside (**50**). **(B)** Lignans with the β-β′/ γ-*O*-γ′ linkage: matairesinol (**51**), wikstromol (**52**), notracheloside (**53**), matairesinol-4,4′-di-*O*-β-D-glucopyranoside (**54**), styraxlignolide D (**55**). **(C)** Lignans with the β-β′/α-*O*-γ′ linkage:lariciresinol (**56**), lariciresinol-4-*O*-β-glucopyranoside (**57**). **(D)** Neolignans: tamariscinoside B (**58**), (*2R*, *3S*)-dihydro-2-(3′,5′-dimethoxy-4′-hydroxyphenyl)-7-methoxy-5-acetyl-benzofuran (**59**), tamariscinoside C (**60**). **(E)** Secolignans: 3,4-trans-3-hydroxymethyl-4-[bis(4-hydroxyphenyl)methyl]butyrolactone (**61**), 2,3-*trans*-3,4-*trans*-2-methoxy-3-hydroxymethyl-4-[bis(4-hydroxyphenyl)methyl]tetrahydrofuran (**62**).

Lignans constitute another group of plant polyphenolics. The core chemical scaffold of lignans are dimeric phenylpropanoid units, including allylphenols and hydroxycinnamyl alcohols and acids, generated through oxidative coupling of radical subunits produced by the actions of laccases or peroxidases (Umezawa, 2003). A number of lignans with shared β-β′/γ-*O*-α′/α-*O*-γ′ (**48**–**50**), β-β′/γ-*O*-γ′ (**51**–**55**), and β-β′/α-*O*-γ′ (**56**–**57**) linkages were identified in *Selaginella* species (Lin et al., 1994; Pan et al., 2001; Feng et al., 2009; Wu and Wang, 2011; **Figures 4A–C**). Notably, compounds **48**–**50** result from dimeric sinapoyl alcohol units, consistent with the finding that *Selaginella* deposits sinapoyl alcohol-derived polymeric syringyl (S) lignin, a lignin type mistakenly thought to be restricted to flowering plants (Towers and Gibbs, 1953; Weng et al., 2008). Indeed, recent structure–function studies showed that *Selaginella* and flowering plants have independently evolved through parallel evolution distinct biosynthetic pathways leading to the biosynthesis of the sinapoyl alcohol monomer (Weng et al., 2010, 2011).

*Selaginella* species also contain neolignans, harboring β-5′ (**58**), or β-5′/α-*O*-4′ (**59**, **60**) linkages (**Figure 4D**; Bi et al., 2004; Zheng et al., 2004b,c; Feng et al., 2008; Wang et al., 2010a). Interestingly, compounds **58**–**60** are dimers of a sinapoyl alcohol unit and either a *p*-hydroxylated *m*-methoxylated acetophenone or a 1-phenylethanol unit. Acetophenone and 1-phenylethanol are major volatile compounds emitted from the flowers of *Camellia sinensis* (Dong et al., 2012). While acetophenone is possibly derived from the general phenylpropanoid pathway through β-oxidation of a β-oxo phenyl propionic acid intermediate, 1-phenylethanol is a reduced product synthesized from acetophenone (Dong et al., 2012). The presence of compounds **58**–**60** in *Selaginella* suggests that *Selaginella* may have acquired or independently evolved through convergent or parallel evolution the ability to synthesize acetophenone and 1-phenylethanol.

A recent study also reported the identification of two unusual secolignans from *S. sinensis* (**61**, **62**; Feng et al., 2009; **Figure 4E**). Secolignans may derive from a matairesinol-type lignan precursor (**51**) through an intramolecular rearrangement that transfers the phenyl group of one monomer unit on to the α-carbon of the second monomer unit. To date, secolignans have only been found in the angiosperm genera *Peperomia* (Monache and Compagnone, 1996), *Justicia* (Kavitha et al., 2003), and *Urtica* (Wang et al., 2008), suggesting yet another case of independent occurrences of similar metabolic traits in distantly related species.

## SELAGINELLINS

**FIGURE 5:**
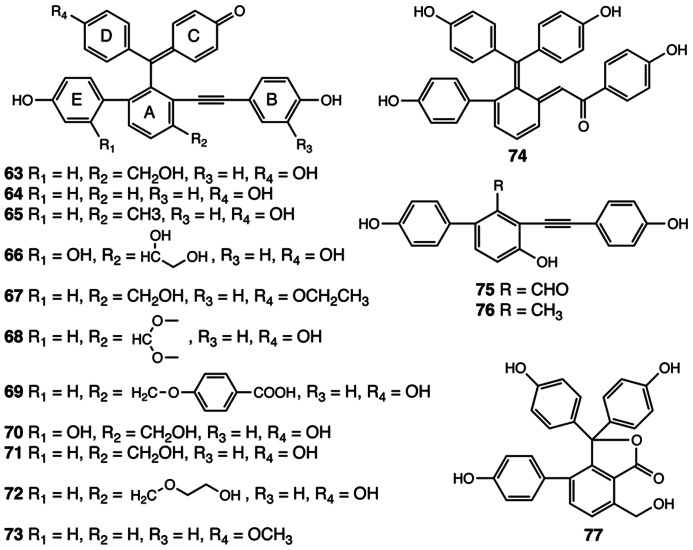
**Selaginellins identified from *Selaginella***. Selaginellin (**63**), Selaginellin A (**64**), Selaginellin B (**65**), Selaginellin C (**66**), Selaginellin D (**67**), Selaginellin E (**68**), Selaginellin F (**69**), Selaginellin I (**70**), Selaginellin J (**71**), Selaginellin M (**72**), Selaginellin N (**73**), Selaginellin G (**74**), Selaginellin K (**75**), Selaginellin L (**76**), Selaginellin H (**77**).

Selaginellins are another group of polyphenolics with a chemical scaffold only found to date in the *Selaginella* genus (**Figure 5**). Selaginellin (**63**), the first member of this compound class, was identified in *S. sinensis* (Zhang et al., 2007). Selaginellin (**63**) was isolated as a racemic mixture, containing a *p*-quinone methide unit and an alkynylphenol moiety (Zhang et al., 2007). Selaginellin undergoes an unusual pH dependent shift of its UV–Vis absorption spectrum thought to serve as a tunable pigment in planta (Zhang et al., 2007). To date, 14 additional compounds structurally related to selaginellin (**64**–**77**) have been identified from *Selaginella* species (Cheng et al., 2008; Tan et al., 2009; Cao et al., 2010a,b; Xu et al., 2011a, b,c; Zhang et al., 2012a; **Figure 5**). Pharmacological studies demonstrate that selaginellin (**63**) confers protective effects on differentiated neuronal cells cultured *in vitro* under different apoptotic conditions, making selaginellins interesting targets for exploring new neuroprotective agents (Wang et al., 2009; Zhang et al., 2012b).

Analogous to taxol in *Taxus brevifolia* (Croteau et al., 2006), selaginellins represent a group of highly elaborated plant natural products, requiring complex multistep metabolic pathways for their biosynthesis. Curiously, in both cases, our current knowledge base suggests that each is restricted taxonomically to only a single genus. Based on the structural information gathered for all the selaginellins identified to date, a putative biosynthetic pathway for selaginellins was advanced recently (Shi et al., 2012). In this proposed pathway, the A ring of selaginellins is derived from orsellinic acid (OA), presumably produced by a structurally basic type III PKS through three decarboxylative condensations using malonyl-CoA on an acetyl CoA starter and a terminating aldol cyclization (Shi et al., 2012). Interestingly, the occurrence of OA has been documented in several fungal species, e.g., *Penicillium madriti* and *Aspergillus nidulans*, wherein OA is produced by structurally complex iterative type I PKSs (Gaucher and Shepherd, 1968; Schroeckh et al., 2009; Sanchez et al., 2010). The functionally analogous overlap of type I and type III PKSs used for the biosynthesis of similar or identical polyketides is not unusual as this is seen for the biosynthesis of tetrahydroxynaphthalene (Austin et al., 2004).

In selaginellins, the E ring, derived from a phenol, is installed onto the OA core through oxidative radical coupling (Shi et al., 2012). The linkage between A and B rings is postulated to form through a benzoin condensation of two phenylaldehyde functional groups, an interesting but often overlooked catalytic reaction probably mediated by thiamin diphosphate-dependent benzaldehyde lyase-type enzymes (Pohl et al., 2002). The resultant benzoin moieties further reduced to a vicinal diol intermediate, followed by dehydration to yield selaginellin L (**76**), carrying the signature alkynyl group. The C and D rings are then added to the core skeleton through additional radical coupling reactions, which, accompanied by other modifications, gives rise to structurally diverse selaginellins (Shi et al., 2012). Selaginellin H (**77**), reported from *S. tamariscina*, is proposed to be derived from OA through a similar mechanism, but without installation of the B ring (Cao et al., 2010b; Shi et al., 2012). Following C and D ring insertion, lactonization gives rise to the 5-membered lactone ring (Shi et al., 2012).

## OTHER PHENOLICS

**FIGURE 6:**
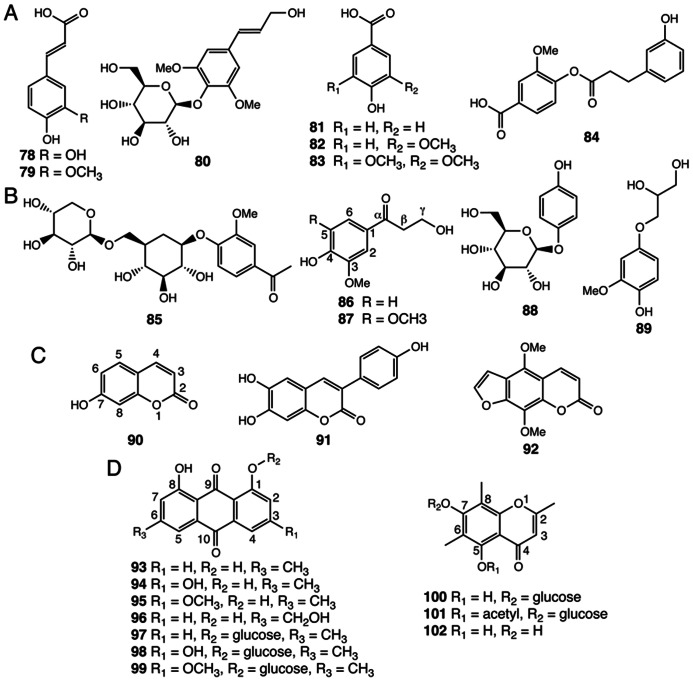
**Other phenolic compounds identified from *Selaginella***. **(A)** Simple phenylpropanoids: caffeic acid (**78**), ferulic acid (**79**), syringin (**80**). Benzenoids: 4-hydroxybenzoic acid (**81**), vanillic acid (**82**), syringic acid (**83**), tamariscina ester A (**84**). **(B)** Uncommon phenylpropanoid derivatives: neolloydosin (**85**), 3-hydroxy-1-(4-hydroxy-3-methoxyphenyl)propan-1-one (**86**), 3-hydroxy-1-(4-hydroxy-3,5-dimethoxyphenyl)propan-1-one (**87**), arbutin (**88**), 1-(4′-hydroxy-3′-methoxyphenyl)glycerol (**89**). **(C)** Coumarins: umbelliferone (**90**), 3-(4-hydroxyphenyl)-6,7-dihydroxycoumarin (**91**), isopimpinellin (**92**). **(D)** Anthraquinones and chromones: chrysophanol (**93**), emodin (**94**), physcion (**95**), aloe-emodin (**96**), chrysophanol-8-*O*-glucoside (**97**), emodin-8-*O*-glucoside (**98**), physcion-8-*O*-glucoside (**99**), uncinoside A (**100**), uncinoside B (**101**), and 8-methyleugenitol (**102**).

Simple phenylpropanoids, such as caffeic acid (**78**), ferulic acid (**79**), and syringin (**80**) have been isolated from *Selaginella* species (**Figure 6A**; Bi et al., 2004; Feng et al., 2009; Xu et al., 2011a). Benzenoids, including ring-substituted benzoic acids and their derivatives (**81**–**84**), were also identified in *Selaginella* (**Figure 6A**; Bi et al., 2004; Feng et al., 2008). The β-oxidative pathway for benzenoid biosynthesis from cinnamic acid to benzoyl-CoA was recently resolved in petunia, and encompasses four reactions catalyzed by three peroxisome-localized enzymes: cinnamate-CoA ligase (CNL), cinnamoyl-CoA hydratase-dehydrogenase (CHD), and 3-ketoacyl-CoA thiolase (KAT; Van Moerkercke et al., 2009; Klempien et al., 2012; Qualley et al., 2012). Homology searches readily identified highly conserved homologs of these three enzymes from both *S. moellendorffii* and *P. patens*, suggesting that the core β-oxidative pathway in benzenoid metabolism is conserved among vascular and non-vascular land plants.

Tamariscina ester A (**84**), a phenolic ester uniquely associated with the *Selaginella* genus (Bi et al., 2004), is derived from acylation of the *p*-hydroxyl group of vanillic acid using an activated *m*-hydroxylated hydrocinnamic acid derivative. The majority of the acylation reactions in plant specialized metabolism characterized to date are catalyzed by enzymes belonging the plant BAHD acyltransferase family (D’Auria, 2006). Comparative genomics suggests that the plant BAHD family radiated extensively but in a parallel fashion in the lycophyte and flowering plant lineages, respectively (Banks et al., 2011). The presence of putative BAHD acyltransferases in *S. moellendorffii* hints that a specialized BAHD acyltransferase for tamariscina ester A (**84**) biosynthesis exists in *Selaginella* species.

*Selaginella* also contains a number of rare phenolic natural products (**85**–**89**; Lin et al., 1994; Bi et al., 2004; Zheng et al., 2004b; Feng et al., 2009), apparently derived from general phenylpropanoids via regiospecific modifications (**Figure 6B**). Neolloydosin (**85**) is a 1-acetophenone-derived diglycoside. Notably, the same 1-acetophenone moiety of neolloydosin (**85**) also resides in a previously discussed lignan (**59**; **Figure 4D**). Compounds **86** and **87**, typified by an α-keto group in the 3-carbon side chain, are most likely biosynthesized from hydroxycinnamyl alcohols via regiospecific hydration followed by oxidation of the olefinic 3-carbon side chain.

Compounds **88** and **89** are hydroquinone natural products. Notably, arbutin (**88**) was previously identified in members of the Ericaceae family (heathers) as well as in pear (Grisdale and Towers, 1960). In *Pyrus* L. (pear), feeding of radioactive tracers suggests that arbutin (**88**) derives from the general phenylpropanoid pathway (Grisdale and Towers, 1960).

Three coumarins, **90**–**92**, were identified from several *Selaginella* species (**Figure 6C**; Chen and Yu, 1986; Bi et al., 2004; Liu et al., 2010). In flowering plants, the coumarin backbone is biosynthesized from phenylpropanoid precursors through aromatic ring *o*-hydroxylation followed by facile lactonization (Kai et al., 2008). The presence of coumarins not only in *Selaginella* but also in bryophytes (Scher et al., 2004) suggests that coumarin biosynthesis may be ancient, and therefore, widely distributed in land plants. It is worth mentioning that isopimpinellin (**92**), an anticarcinogenic furanocoumarin found in *S. moellendorffii* (Chen and Yu, 1986), was previously reported to be sporadically distributed in flowering plants, including citrus, sweet potato, and members of the Umbelliferae, alternatively Apiaceae (carrot and parsley) family (Minamikawa et al., 1963; Gopälakrishna et al., 1977; Kleiner et al., 2002). In Umbelliferae, isopimpinellin (**92**) is metabolized from umbelliferone (**90**) through seven enzymatic steps, including 6-prenylation, oxidation-mediated furan formation, and regiospecific aromatic ring hydroxylations followed by two *O*-methylation reactions (Larbat et al., 2007, 2009). Based upon gene comparisons, *Selaginella* lacks obvious homologs of the CYP71-family P450s as well as the OMT required for isopimpinellin biosynthesis in Queen Anne’s lace (*Ammi majus*), implying that the elaborated isopimpinellin biosynthetic pathway may have emerged independently through convergent and/or parallel evolution in *Selaginella* and flowering plants (Hehmann et al., 2004; Banks et al., 2011).

Seven anthraquinones (**93**–**99**) and three chromones (**100**–**102**) were identified in *Selaginella* (Ma et al., 2003; Yang et al., 2011; **Figure 6D**). Naturally occurring anthraquinones were previously reported in multiple families of flowering plants, including Aloeaceae (Demian and Gripshover, 1989), Hypericaceae (Karppinen et al., 2008), Rhamnaceae (van den Berg and Labadie, 1984), and Rubiaceae (Burnett and Thomson, 1968), as well as in a number of fungal species, such as *A. nidulans* (Chiang et al., 2010) and a endophytic fungus isolated from *Hypericum perforatum* (Kusari et al., 2008).

Chromones, which are structurally related to anthraquinones, were found not only in flowering plants notably in Aloeaceae (Hutter et al., 1996), Umbelliferae (Gui et al., 2011), and Cunoniaceae (Tschesche et al., 1979) species, but also in a lichen fungal symbiont *Lecanora rupicola* (Fox and Huneck, 1969). Unlike the biosynthesis of anthraquinones in fungi, where the octaketide backbone is generated by iterative type I PKSs (Chiang et al., 2010), plants employ much simpler and convergently-derived type III PKSs to synthesize similar polyketide-based skeletons (Austin et al., 2004). In *Aloe arborescens*, two highly similar type III PKSs (>90% protein sequence identity) exhibit distinct biochemical functions as octaketide synthase (OKS) and pentaketide chromone synthase (PCS) en route to anthraquinone and chromone biosynthesis, respectively (Abe and Morita, 2010; **Figure 2B**). Mechanistically, a single residue polymorphism, which modulates the volume of the active site available to the polyketide elongation and cyclization reactions, directs the polyketide chain length selection of *Aloe arborescens* OKS and PCS (Morita et al., 2007). Moreover, functional OKSs were also identified in *H. perforatum*, although they are not closely related to *A. arborescens* OKS (Bais et al., 2003; Karppinen et al., 2008) again suggestive of parallel evolution from a more distantly related type III PKS ancestor.

*Selaginella* also lacks a clear homolog of angiosperm OKSs and PCSs, suggesting the occurrences of anthraquinone and chromone biosynthesis in plants and fungi are highly polyphyletic. It is worth mentioning that the three chromones (**100**–**102**) isolated from *Selaginella* also carry the unusual 6- and 8-carbon methyl groups, reminiscent of the two C-methylations on the equivalent aromatic ring carbons of α-tocopherol (DellaPenna and Pogson, 2006). The specialized *C*-methyltransferases involved in chromone biosynthesis in *Selaginella* may be evolutionarily related to the 2-methyl-6-phytylplastoquinol methyltransferase and the γ-tocopherol methyltransferase critical to plant vitamin E biosynthesis (DellaPenna and Pogson, 2006).

## ALKALOIDS

**FIGURE 7:**
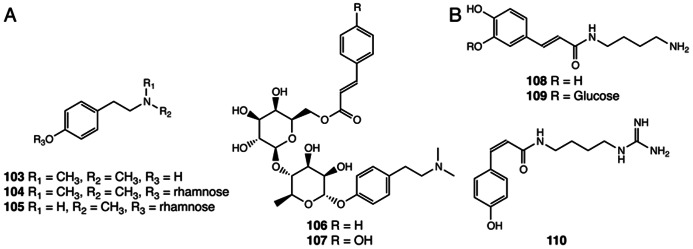
**Alkaloids identified from *Selaginella***. **(A)** *N*-methyltyramine derivatives: hordenine (**103**), hordenine-*O*-α-rhamnopyranoside (**104**), *N*-methyltyramine-*O*-α-rhamnopyranoside (**105**), hordenine-*O*-[(6-*O*-cinnamoyl)-*O*-β-glucopyranosyl]-α-rhamnopyranoside (**106**), hordenine-*O*-[(6-*O*-*p*-coumaroyl)-*O*-β-glucopyranosyl]-α-rhamnopyranoside (**107**). **(B)** Hydroxycinnamoyl polyamine alkaloids: paucine (**108**), paucine 3′-β-D-glucopyranoside (**109**), N^1^-*cis*-*p*-coumaroylagmatine (**110**).

Alkaloids are nitrogen-containing natural products widely distributed in nature (Roberts and Wink, 1998). This class of specialized compounds often exhibit significant pharmacological and psychoactive effects and are widely used as medicines and mood modulators (Facchini, 2001). Five *N*-methyltyramine-derived phenolic alkaloids were reported in *S. doederleinii* (**103**–**107**; Chao et al., 1987, 1990; Lin et al., 1991; **Figure 7A**). The first committed step for *N*-methyltyramine biosynthesis from tyrosine to tyramine is catalyzed by tyrosine decarboxylase, gene homologs of which can be found in the *S. moellendorffii* and *P. patens* genomes (Kawalleck et al., 1993). It was shown that hordenine (**103**), a compound first discovered in barley, is biosynthesized by the step-wise *N*-methylation of tyramine in barley, although genes encoding specific tyramine *N*-methyltransferases are yet to be identified (Mann and Mudd, 1963).

Three hydroxycinnamoyl polyamine alkaloids were isolated from *S. moellendorffii* (**108**–**110**), two of which were previously reported in angiosperm species (**Figure 7B**). Paucine (**108**) is found in *Nicotiana* species acting as a stress-inducible metabolite (Mizukasi et al., 1971; Onkokesung et al., 2011), and *N*^1^-*cis*-*p*-coumaroylagmatine (**110**) is found in the nyctinastic plant *Albizia julibrissin* and plays a role as a leaf-opening signal (Ueda et al., 1997).

The biosynthesis of hydroxycinnamoyl polyamines in flowering plants requires specialized acyltransferases that shift the hydroxycinnamoyl moiety from a hydroxycinnamoyl-CoA to distinct acyl acceptor polyamines (Burhenne et al., 2003; Muroi et al., 2009; Onkokesung et al., 2011). Interestingly, two acyltransferases, belonging to different phylogenetic clades of the BAHD family (Banks et al., 2011), are found in *Arabidopsis* and barley, respectively, that, in a parallel fashion, result in the same biochemical outcome. Both encode functional *p*-coumaroyl-CoA:agmatine *N*^1^-*p*-coumaroyltransferases involved in *N*^1^-*trans*-*p*-coumaroylagmatine biosynthesis (Burhenne et al., 2003; Muroi et al., 2009). *Selaginella* lacks homologous systems for any of the known angiosperm polyamine *N*-hydroxycinnamoyltransferases, and, presumably exploits highly divergent acyltransferases or an analogous biosynthetic system to catalyze the same reactions. The accumulation of the *cis*-isomer of *p*-coumaroylagmatine in *S. moellendorffii* and *A. julibrissin* further implies the existence of *p*-coumaroyl *trans*–*cis* isomerases in these species. This type of *trans*–*cis* isomerase is currently an undefined biosynthetic activity, which is also suggested to be critical in the biosynthesis of *cis*-coumarinic acid-β-D-glucoside in *Melilotus alba* (Stoker, 1964).

## TERPENOIDS

Terpenoids, alternatively isoprenoids, possess hydrocarbon cores originating with the linkage of 5-carbon isoprene units (Russell and Cohn, 2012). As a compound class, terpenoids exist in all three domains of life (Gershenzon and Dudareva, 2007). In many organisms, terpenoids are biosynthesized using the mevalonic acid (MVA) pathway, while in plant plastids and many organisms in the bacteria domain, terpenoids are also biosynthesized using the 2-*C*-methyl-D-erythritol 4-phosphate/1-deoxy-D-xylulose 5-phosphate (MEP/DXP) pathway (Kuzuyama, 2002). In plants, extended terpenoids, including carotenoids (eight isoprene units – 40-carbons) and steroids (six isoprene units – 30-carbons), are often associated with primary metabolism, whereas an enormous diversity of terpenoids synthesized by plants are categorized as specialized metabolites (Chen et al., 2011).

**FIGURE 8:**
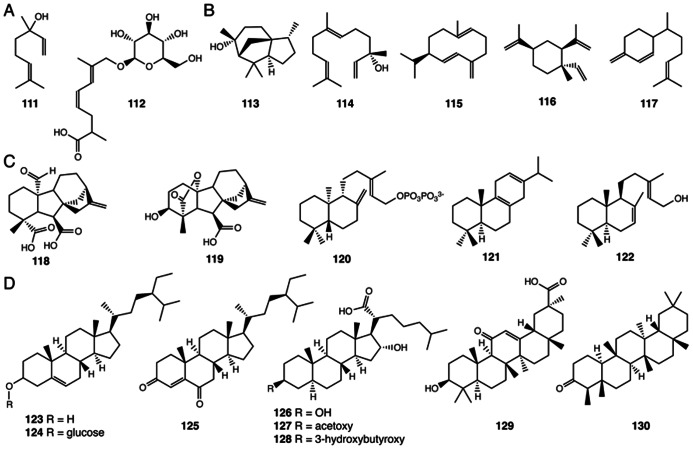
**Terpenoids identified from *Selaginella***. **(A)** Monoterpenes: linalool (**111**), (4*Z*,6*E*)-2,7-dimethyl-8-hydroxyocta-4,6-dienoic acid 8-*O*-β-D-glucopyranoside (**112**). **(B)** Sesquiterpenes: cedrol (**113**), (+)-(*3S*)-nerolidol (**114**), (+)-germacrene D (**115**), (-)-β-elemene (**116**), **β**-sesquiphellandrene (**117**). **(C)** Diterpenes: gibberellin A4 (**118**), gibberellin A24 (**119**), *ent*-copalyl diphosphate (**120**), miltiradiene (**121**), λ-7,13e-dien-15-ol (**122**). **(D)** Triterpenes: β-sitosterol (**123**), β-daucosterin (**124**), pulvinatadione (**125**), 3β,16̩-dihydroxy-5〈,17^®^-cholestan-21-carboxylic acid (**126**), 3^®^-acetoxy-16〈-hydroxy-5α, 17β-cholestan-21-carboxylic acid (**127**), 3β-(3-hydroxybutyroxy)-16α-hydroxy-5α, 17β-cholestan-21-carboxylic acid (**128**), glycyrrhetinic acid (**129**), friedelin (**130**).

Two monoterpenes (two isoprene units – 10-carbons – **111**, **112**) and five sesquiterpenes (three isoprene units – 15-carbons – **113**–**117**) have been found in *Selaginella* (Hui et al., 2005; Wang et al., 2011; Li et al., 2012; **Figures 8A, B**) and other plant lineages. Monoterpenes and sesquiterpenes constitute an important class of volatile, semi-volatile, and non-volatile hydrocarbon and biosynthetically elaborated compounds produced by plants. These chemicals serve as important modulators of interspecies interactions including attraction of pollinators and seed dispersers as well as chemical defenses against pathogens and herbivores (Dudareva et al., 2004). Since *Selaginella* does not reproduce through flowers and seeds, the biological roles of monoterpenes and sesquiterpenes of *Selaginella* remain unresolved.

In seed plants, monoterpenes and sesquiterpenes are biosynthesized from geranyl pyrophosphate (10-carbon – GPP) and farnesyl pyrophosphate (15-carbon – FPP), respectively, through the catalytic action of monoterpene and sesquiterpene synthases, respectively. Phylogenetic analyses of plant terpene synthases (TPSs), including mono-, sesqui-, and diterpene synthases, suggest that the monoterpene and sesquiterpene synthases are evolutionarily derived from the more conserved diterpene (20-carbon) synthases (Bohlmann et al., 1998). It was recently reported that the *S. moellendorffii* genome encodes 66 TPSs, among which, 18 TPSs are homologous to the canonical plant diterpene synthase genes (Banks et al., 2011; Li et al., 2012).

Paradoxically, the remaining 48 TPSs share little sequence similarity to plant TPSs, and, are in fact, more closely related to bacterial TPS genes (Li et al., 2012). The bacterial TPSs are structurally related to the α-domain of plant TPSs despite a lack of sequence similarity (Li et al., 2012). Functional analyses of the *Selaginella* bacterial-like TPSs further demonstrated that 6 of the 48 bacterial-like TPSs act as monoterpene and sesquiterpene synthases *in vitro* with varying levels of catalytic promiscuity. To date, the functionally characterized TPSs produce terpenes matching those emitted from *Selaginella* plants (Li et al., 2012). This genomic discovery illustrates a remarkable case of parallel origins of monoterpene and sesquiterpene biosynthesis in terrestrial plants. Lycophytes, such as *S. moellendorffii*, appear to have recruited an ancestral bacterial-type TPS via horizontal gene transfer, and, through gene duplication followed by neofunctionalization, these newly acquired biosynthetic activities exploited the catalytic landscape of this unique TPS clade (Li et al., 2012).

Diterpene gibberellins (GAs) are a family of 20-carbon tetracyclic diterpenoids, which function as phytohormones regulating many aspects of plant growth and development (Yamaguchi, 2008). Bioactive GA_4_ (**118**) and GA_24_ (**119**) were identified in *S. moellendorffii* as the major GA species (Hirano et al., 2007; **Figure 8C**). This observation is consistent with subsequent phylogenetic analyses suggesting that the early steps of GA biosynthesis from geranylgeranyl (20-carbon) pyrophosphate (GGPP) to GA_12_ are conserved in *Selaginella*, ferns and seed plants (Banks et al., 2011). Among the 18 canonical diterpene synthase-like genes identified in the *S. moellendorffii* genome, four have been biochemically characterized. Two, *Sm*TPS9 and *Sm*TPS10, exhibit monofunctional diterpene synthase activities, converting GGPP to *ent*-copalyl diphosphate (**120**; Li et al., 2012). *Sm*MDS and *Sm*CPSKSL1 are bifunctional diterpene synthases that produce miltiradiene (**121**) and λ-7,13E-dien-15-ol (**122**), respectively, from GGPP (Mafu et al., 2011; Sugai et al., 2011; **Figure 8C**).

Eight triterpenes (six isoprene units – **123**–**130**) have been isolated from *Selaginella* species (Tan et al., 2004; Gao et al., 2007; Yang et al., 2010; **Figure 8D**). Tetracyclic pulvinatadione (**125**), first reported in *S. pulvinata* (Tan et al., 2004), is likely metabolized from the more widely distributed β-sitosterol (**123**; De-Eknamkul and Potduang, 2003). Tetracyclic sterols **126**–**128** have been isolated from *S. tamariscina* (Gao et al., 2007).

The pentacyclic glycyrrhetinic acid (**129**) was isolated from *S. delicatula* (Yang et al., 2010). Glycyrrhetinic acid (**129**) is the aglycone core of glycyrrhizin, an economically important sweet-tasting compound originally isolated from licorice (Seki et al., 2011). The biosynthesis of glycyrrhetinic acid (**129**) in licorice requires two specialized cytochrome P450s belonging to the CYP72 and CYP88 families. Each catalyzes regiospecific oxidation reactions of the triterpene β-amyrin core (Seki et al., 2011). As no obvious homolog of either of these enzymes is found in the *S. moellendorffii* genome, the equivalent biochemical activities likely emerged independently as homologous or analogous enzymes in the *Selaginella* genus through parallel or convergent evolution, respectively.

The pentacyclic friedelin (**130**) was also identified from *S. delicatula* (Yang et al., 2010). Mechanistically, friedelin (**130**) is a remarkably rearranged pentacyclic triterpene originating from a proton-activated oxidosqualene catalytic intermediate bound in the active site of an oxidosqualene cyclase. The unusual pentacyclic structure results from the concerted rearrangement of carbocationic catalytic states (Kurti et al., 2008). Recently, a specialized oxidosqualene cyclase capable of converting oxidosqualene to friedelin (**130**) was identified in *Kalanchoe daigremontiana* (Wang et al., 2010b). While *S. delicatula* produces friedelin (**130**), the *S. moellendorffii* genome does not contain obvious homologs encoding to the *K. daigremontiana* enzyme, again suggesting that the equivalent biochemical activity likely emerged independently, through either parallel or convergent means in the *Selaginella* genus.

## INDEPENDENT RADIATION OF SPECIALIZED METABOLIC ENZYME FAMILIES

The recent availability of the whole-genome sequence of the bryophyte *P. patens* and now the lycophyte *S. moellendorffii* fills critical gaps in our genome-level understanding of the molecular evolution and radiation of the green plant lineage. Together with previous genomes of seed plants and algae, these newly available genomic resources greatly accelerate our ability to carry out genome-wide comparisons of gene families spanning the entire green plant lineage (Bowman et al., 2007). Large-scale phylogenetic analyses of several enzyme families deeply rooted in plant specialized metabolism, e.g., the cytochrome P450s, BAHD acyltransferases, TPSs, OMTs, polyphenol oxidases, and glycosyltransferases (GTs), reveal a consistent evolutionary progression. This trend suggests that ancestral vascular plants probably contained a relatively small biochemical repertoire of catalytic machineries, which then underwent extensive lineage-specific, and often independent expansion in lycopodiophytes and euphyllophytes (Banks et al., 2011; Weng et al., 2011; Harholt et al., 2012; Li et al., 2012; Tran et al., 2012).

Unexpectedly, the resultant rich chemical diversity in lycopodiophytes, as evidenced from metabolite isolation across the *Selaginella* genus, rivals that for developmentally more complex species in the seed plant lineages. Since lycopodiophytes and euphyllophytes have co-existed on earth for the last 400 million years, often occupying similar global habitats, highly similar metabolic traits, represented by chemicals of specialized metabolism, repeatedly emerged in the two lineages through parallel and convergent evolution possibly prompted by overlapping selective pressures. This ancestral enzyme independence is likely more common than previously thought. Using a combination of computational, structural, genomic, metabolomic, and biochemical tools, we are now able to piece together a fascinating example of independent adaptive strategies for survival and fitness at the molecular, organismal, and ecological levels driving phenotypic convergence in the evolutionarily diverse green plant lineage.

## FUTURE PERSPECTIVES

Our expanding understanding of chemodiversity in the *Selaginella* genus, a genus that parted ways with the more well-studied seed plants 400 million years ago, sets the stage for uncovering the divergent and convergent restraints governing enzyme and metabolic evolution over defined time periods. Moreover, by combining this information with knowledge of the chemical and ecological restraints shaping plant adaptation to the biotic and abiotic factors impinging on ecosystems, we can learn from the past and look forward to a future where we can more predictably enhance plant adaptation in the face of global climate change. A systems-level approach that integrates multiple pieces of information, including transcriptomic data and metabolomic profiles of specialized metabolites across different tissue types of a single *Selaginella* species or related *Selaginella* species inhabiting disparate natural environments, will accelerate candidate gene identifications responsible for particular metabolic traits.

Currently, *in vitro* biochemistry and transgenic expression of *Selaginella* genes in model plants, such as *Arabidopsis* and rice, have been the primary approaches for functional characterization of *Selaginella* enzymes. The development of effective transformation techniques in *Selaginella* species that allow gene knock-in, knock-out, and knock-down will move *Selaginella* from a reference genus to model species. These developments will afford rapid elucidation of the genetic basis of specialized metabolism in *Selaginella*, and by comparison to other members of the green plant lineage, a more comprehensive and predictive understanding of plant chemoadaptation.

Comparative biochemical and structural analyses of functionally analogous and homologous enzymes of independent origins in *Selaginella* and higher plants will also illuminate mechanistic restraints that guide similar or disparate mutational trajectories ultimately intersecting on nearly identical metabolic outcomes. This type of information will generally enrich our understanding of the emergence and ongoing evolution of new and existing catalytic strategies in nature through divergent, parallel and convergent evolution of sessile organisms so dependent on chemodiversity as a response to a myriad of global ecologies. It is not too early to predict that by understanding the chemical strategies used by plants to adapt to environmental challenges, we will provide predictable and sustainable tools for engineering more productive crops, for accelerating drug discovery and for generating biorenewable chemicals and fuels.

## Conflict of Interest Statement

The authors declare that the research was conducted in the absence of any commercial or financial relationships that could be construed as a potential conflict of interest.
